# Characterization and functional prediction of the dental plaque microbiome in patients with alveolar clefts

**DOI:** 10.3389/fcimb.2024.1361206

**Published:** 2024-05-10

**Authors:** Yuehua Zhang, Qiang Zhi, Jiajun Shi, Zehua Jin, Zhuojun Zhou, Zhenqi Chen

**Affiliations:** ^1^ Department of Orthodontics, Shanghai Ninth People’s Hospital, Shanghai Jiao Tong University School of Medicine, Shanghai, China; ^2^ College of Stomatology, Shanghai Jiao Tong University, National Center for Stomatology, National Clinical Research Center for Oral Diseases, Shanghai Key Laboratory of Stomatology, Shanghai Research Institute of Stomatology, Shanghai, China; ^3^ Department of Implant Dentistry, Shanghai Ninth People’s Hospital, Shanghai Jiao Tong University School of Medicine, Shanghai, China; ^4^ Department of Stomatology, Tongji Hospital, School of Medicine, Tongji University, Shanghai, China; ^5^ Department of General Dentistry, Shanghai Ninth People’s Hospital, Shanghai Jiao Tong University School of Medicine, Shanghai, China

**Keywords:** alveolar cleft, alveolar bone grafting, supragingival plaque microorganisms, 16S rRNA sequencing, microbial diversity

## Abstract

**Introduction:**

Alveolar cleft (AC) is a common congenital defect in people with cleft lip and palate (CLP). Alveolar bone grafting (ABG) is typically performed during adolescence, resulting in the fissure remaining in the mouth for a longer length of time. Patients with AC have a greater rate of oral diseases such as dental caries than the normal population, and the precise characteristics of the bacterial alterations caused by AC are unknown.

**Methods:**

We recruited a total of 87 subjects and collected dental plaque samples from AC adolescents (AAP), post-operative ABG adolescents (PAP), healthy control adolescents (CAP), AC young adults (AYP), post-operative ABG young adults (PYP), and healthy control young adults (CYP). The sequencing of 16S rRNA genes was performed.

**Results:**

The microbial composition of plaque from alveolar cleft patients differed significantly from age-matched healthy controls. Linear discriminant analysis effect size (LEfSe) analysis revealed that AAP was enriched for *Neisseria, Haemophilus, Fusobacterium, Rhodococcus, Aggregatibacter, Gemella*, and *Porphyromonas*, whereas AYP was enriched for *Capnocytophaga, Rhodococcus*, and *Actinomyces-f0332*. There were phenotypic differences in facultatively anaerobic, Gram-negative, Gram-positive, and oxidative stress tolerance between the AYP group with longer alveolar cleft and the healthy control group according to Bugbase phenotypic predictions. Alveolar bone grafting did not alter the functional phenotype of alveolar cleft patients but reduced the number of differential genera between alveolar cleft patients and healthy controls at both ages.

**Conclusions:**

Our study systematically characterized the supragingival plaque microbiota of alveolar cleft patients, post-alveolar bone grafting patients, and matched healthy controls in two ages to gain a better understanding of plaque ecology and microbiology associated with alveolar clefts.

## 1 Introduction

Non-syndromic cleft lip with or without cleft palate (NSCL/P) is the most common congenital anomaly affecting the oral and maxillofacial region. It arises during embryogenesis due to a breakdown in tissue fusion, which also encompasses the alveolar cleft (Da Silva et al., 2023). As the frontonasal prominence develops, an inadequate union of the maxillary and median nasal processes results in the formation of the alveolar cleft (AC) (Mundra et al., 2022). Approximately 75% of all complete cleft lip and palate (CLP) patients are AC patients who require alveolar bone grafting (ABG) (Bangun et al., 2023; Destrez et al., 2023). AC, which is situated in the region bounded by the lateral incisors and canines, causes pathologic phonation, orofacial fistulas, fluid reflux, overcrowding, maxillary anterior-posterior and transverse defects, absence of bony support for maxillary anterior teeth, and disruption of the continuity of the maxillary arch (Gaillot et al., 2007; Guo et al., 2011; Tamasas and Cox, 2017; Ahmed et al., 2020; Buile et al., 2022).

By facilitating cuspid migration and eruption via the cancellous bone, closing oro-nasal fistulas, and enhancing oral hygiene, alveolar bone grafting (ABG) can be utilized to effectively correct these abnormalities (Patmon et al., 2023). Presently, the mixed dentition period in adolescents, mainly between the ages of 9 and 12, is the best time interval for alveolar bone grafting (Kim and Jeong, 2022). Additionally, it is frequently advised that the procedure be executed near the midpoint of the canines’ root development (Ferguson et al., 2023). To increase surgical success, cancellous bone from the iliac crest is the most frequently used bone graft material (Kang, 2017). ABG provides bone support for the subsequent eruption of permanent teeth. Subsequent orthodontic treatment moves the permanent teeth into the bone graft area, improving anterior esthetics and occlusion. At the same time, the continuity of the maxillary arch after ABG is necessary for maxillary anterior traction during subsequent orthodontic treatment, which is optimal during the adolescent years.

In people with cleft lip and palate, it is recognized that improper connection between the lip and palate causes changes in the typical flora of these two regions (Machorowska-Pieniążek et al., 2017; Arboleda et al., 2023; Chen et al., 2023; Świtała et al., 2023). Numerous studies have shown an increased frequency of potentially pathogenic fungal and bacterial colonization in the normal oral microbiome of cleft lip and palate patients compared to healthy controls, particularly *Candida species*, *Staphylococcus aureus*, *Lactobacilli* and *Streptococcus mutans* (Tuna et al., 2008; Jin et al., 2016; Silva et al., 2018; Machorowska-Pieniążek et al., 2021; Alansari et al., 2023). And these fungal and bacterial may be associated with a variety of oral diseases and postoperative complications of cleft lip and palate repair in CLP patients. Furthermore, these patients have considerably elevated plaque indices and are more prone to developing caries and periodontal disease in comparison to healthy controls (May et al., 2023; Surtie et al., 2023). Infections produced by bacteria may also be more prevalent among people with CLP, according to additional research. Oral, nasal, pharyngeal, and ear fissures, as well as bodily fluids, secretions, and excretions, are all potential sites for diseases (Lee et al., 2021; Rigotti et al., 2022). According to research, maintaining the stability of microbiota is of the utmost importance to prevent dysbiosis (Zaura et al., 2014), which may cause disorders such as dental caries or periodontitis to develop from healthy conditions. In a recent study, by evaluating critical signaling molecules produced by oral bacteria, biosynthetic gene clusters (BGCs) of the human oral microbiome have been identified (Aleti et al., 2019). Significant heterogeneity between health and disease is a consequence of oral microbiota dysbiosis at the local level. In the oral microbiome, plaque microbial diversity and dysregulated interactions are crucial in the etiology of dental caries (Fine and Schreiner, 2023; Parga et al., 2023; Shao et al., 2023). Studies on the ecology of dental plaque in cleft lip and palate patients at different stages of the disease may help to provide new perspectives and approaches for a better understanding of cleft lip and palate patients and thus for treatment and control methods of their oral diseases.

Oral diseases such as dental caries are commonly observed in individuals with cleft lip and palate. However, a significant proportion of these patients had previously experienced cleft lip and palate repair during infancy, possess solely an alveolar cleft, or have undergone alveolar bone grafting. Oral microbiology research in patients with cleft lip and palate has focused primarily on newborns and young children who have not undergone cleft lip and palate repair. The oral microbiome of AC and post-operative ABG patients, including adolescents and young adults, has been the subject of less research.

**Figure 1:**
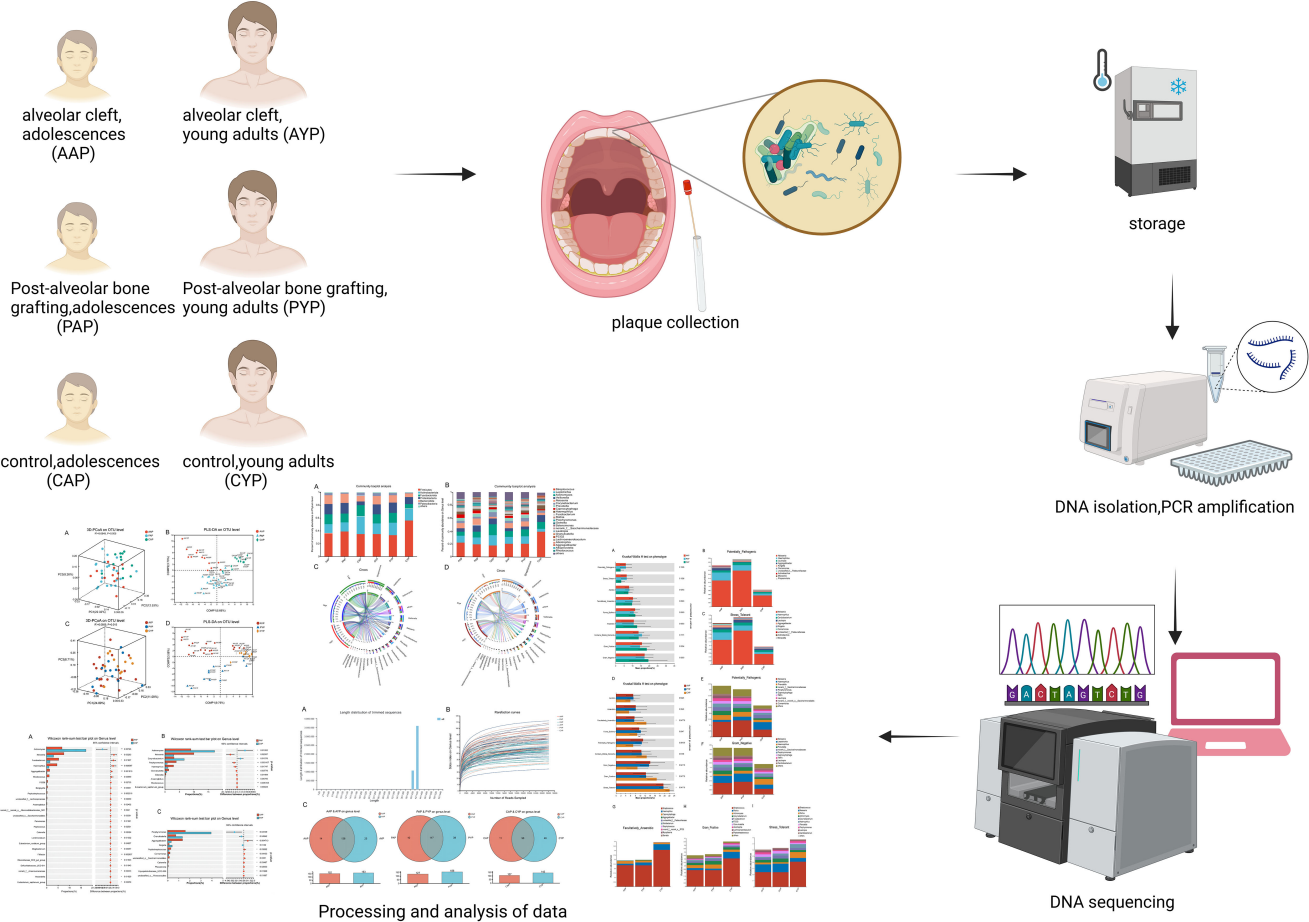
High-throughput sequencing and bioinformatics analysis of dental plaque between three groups of two ages.

We examined the dental plaque microbiota of healthy controls, AC patients, and patients who had undergone ABG. This research examined adolescents and young adults as participants. It was postulated that the intricate interplay of bacteria in individuals diagnosed with AC may play a role in the progression of oral disease. The objective of this research was to use the V3-V4 region of the 16S rRNA gene to identify the supragingival plaque bacteria in patients with AC, and to predict the bacterial phenotype in these patients (**Figure 1**).

## 2 Method

### 2.1 Study participants

This study was conducted following the Declaration of Helsinki. It was approved by the Ethics Committee of Shanghai Ninth People’s Hospital, Affiliated with Shanghai Jiao Tong University School of Medicine (Approval number: SH9H-2022-T347-2). Informed consent was provided to and signed by every participant. 87 individuals who were admitted to the Department of Orthodontics and the Department of Oral and Maxillofacial Surgery at the Ninth People’s Hospital, which is affiliated with Shanghai Jiaotong University School of Medicine, comprised the study participants. These included AC adolescents, post-operative ABG adolescents, healthy control adolescents, AC young adults, post-operative ABG young adults, and healthy control young adults. Diagnosed as having non-syndromic complete unilateral cleft lip and palate (UCLP), adolescents and young adults with AC belonged into the respective age range (6–18 and 19–28 years), had cleft lip and palate repair performed at the age of less than two years old, and had not had alveolar bone grafting (ABG). In the selection of the age group of subjects, according to the age segmentation established by the World Health Organization, adolescence is 9-17 years. Currently, mixed dentition in adolescents, mainly between 9 and 12 years of age, is the best age range for alveolar bone grafting. Meanwhile, early alveolar bone grafting is also being explored for children aged 6 to 8 years. For ease of delineation, we refer to children and adolescents aged 6-18 years as the adolescent period for comparison with the young adult period of 19-28 years when ABG is deferred.

Adolescents and young adults who underwent ABG had comparable AC diagnoses and had undergone ABG surgery at least 6 months before this study without complications. Bone graft outcomes were assessed with cone beam computed tomography (CBCT) to guarantee the grafts’ success. The operation was performed at the Department of Oral Craniomaxillofacial Surgery, Ninth People’s Hospital, which is affiliated with Shanghai Jiao Tong University. Oral or respiratory infections, contagious diseases, generalized systemic disease, prior or current mobile or fixed orthodontic treatment, and complications such as oronasal fistulas or recurrent infections following lip and palate repairs were among the exclusion criteria. Trained investigators obtained primary demographic data from participants and their legal guardians, including age and sex. The dental professional overseeing the procedure provided diagnostic and surgical data. In addition, a brief oral examination of the subjects was performed to document the plaque index and the DMFS (dmfs) index (number of decayed, missing, and filled surfaces).

### 2.2 Sample collection, DNA extraction, and PCR amplification

Sampling of supragingival plaques was conducted as previously described (Gilbert Klaczko et al., 2023). A clinical examination was conducted before sample collection, and participants were instructed to rinse their mouths with normal saline to eliminate any residual food particles. Oral swabs were utilized to collect supragingival plaque samples from the four first incisors and four first molars of each participant after the tooth had been dried and exposed, and then these 8 samples were pooled. The subjects hadn’t had a professional teeth cleaning in the three months before sampling to ensure a sufficient sample size. Following diagnosis and age-based classification, all samples were cooled to -80°C before undergoing subsequent processing.

Plaque-associated total bacterial genomic DNA was isolated from mouth swabs utilizing the QIAamp DNA Mini Kit (Qiagen, Valencia, CA, USA). Using a NanoDrop^®^ ND-2000 spectrophotometer (Thermo Scientific Inc., USA) and 1.0 percent agarose gel electrophoresis, the quality and concentration of the DNA were assessed. The V3–V4 region of the bacterial 16S rRNA gene was amplified using primers 338F and 806R (Liu et al., 2016). The PCR amplification was carried out on an ABI GeneAmp^®^ 9700 PCR thermocycler (ABI, California, USA). TransGen AP221-02, TransStart Fastpfu DNA Polymerase, and 20 μL reaction system were used to carry out the formal PCR experiment. The PCR amplifications were performed with 27 cycles and as previously described (Picchianti-Diamanti et al., 2018). Using the AxyPrep DNA Gel Extraction Kit (Axygen Biosciences, Union City, CA, USA) and Quantus™ Fluorometer (Promega, USA), the amplified PCR product was extracted from a 2 percent agarose gel for detection and purification, amplicon fragment size verification, and quantification after amplification.

### 2.3 Illumina MiSeq sequencing and data processing

Utilizing the Illumina MiSeq PE300 platform (Illumina, San Diego, USA) and standard operating methods, purified amplicons were pooled at equimolar levels, and paired-end sequencing was performed. Using fastp version 0.19.6 (Chen et al., 2018) and FLASH version 1.2.7 (Magoč and Salzberg, 2011), the raw FASTQ files were demultiplexed, quality filtered, and merged using the same standard as described in the previous study (Zhang et al., 2023). After that, the higher-quality sequences were grouped using UPARSE 7.1 (Edgar, 2013) into Operational Taxonomic Units (OTUs) with a uniform 97 percent similarity. To reduce the impact of sequencing depth on the alpha and beta diversity measures, 20,000 rarefied 16S rRNA gene sequences from each sample were obtained, which still resulted in an average Good’s coverage of 99.09 percent, respectively. With a confidence level of 0.7, the classification of each OTU representative sequence was evaluated using RDP Classifier version 2.2 (Wang et al., 2007) against the 16S rRNA gene database (Silva v138). Then, OTU taxonomic analyses retained the domain level of bacteria for OTUs identified to at least the phylum level, and removed OTUs that may have amplification or sequencing errors that were identified as bacteria only at the domain level. And remove unwanted lineages such as chloroplasts, mitochondria, archaea, and eukaryotes. The data presented in the study were deposited in the SRA repository (NCBI). The accession number of the datebase is PRJNA 1062077.

### 2.4 Statistical analysis

OTU dataset and Mothur v1.30.2 (Schloss et al., 2009) were used to compute rarefaction curves based on species and genus level. Alpha diversity was assessed using the Chao, Ace, Simpson, and Shannon indices. Alpha and beta diversity were analyzed at the genus level. The software package Vegan v2.5-3 was utilized to perform weighted-normalized-unifrac metric-based Principal Coordinate Analysis (PCoA) to identify variations in the microbial community composition across several plaque samples. Using the Vegan v2.5-3 package, the PERMANOVA (ADONIS) test was also carried out to ascertain the statistical significance of each sample group’s percentage of variance explained. To ascertain whether bacterial genera were significantly enriched, linear discriminant analysis (LDA) effect sizes (LEfSe) (Segata et al., 2011) were employed (LDA scores >3, *p* < 0.05). To anticipate the phenotypic function of each plaque sample group, overexpression phenotypes were identified using BugBase (Duan and Li, 2023). Statistical analyses were performed using SPSS 25.0 (IBM Corporation, Armonk, NY, United States). Chi-squared test was used to determine differences in categorical variables of gender between groups. One-way ANOVA and nonparametric Kruskal-Wallis H test were used to determine significant differences between the three groups. FDR correction was applied for multiple testing corrections and post-hoc tests using Scheffe’s test. Non-parametric Wilcoxon rank-sum test was used to analyze the significant difference between the two groups.

## 3 Results

### 3.1 Grouping of the participants and sequencing analysis

Genomic DNA was extracted from supragingival plaque samples, amplified, and sequenced for each of the 87 participants. Plaque was collected from 32 AC patients (12 adolescents or AAP; 20 young adults or AYP), 34 post-operative ABG patients (21 adolescents or PAP; 13 young adults or PYP), and 21 healthy control participants (12 adolescents or CAP; 9 young adults or CYP). There were no significant differences in gender, DMFS (dmfs), or PLI index between adolescents (**Table 1**) and young adults (**Table 2**). **Supplementary Tables S1**, **S2** describe the clinical information for the teenage and young adult individuals separately.

**Table 1:** Descriptive statistics and statistical comparisons between AAP, PAP, and CAP.

	AAP	PAP	CAP	*p*-value
Age, median (IQR)	13.5(11.3-16.8)	12.2(9.0-16.0)	12.0(10.3-14.0)	0.409^2^
Gender				0.364^1^
male, n (%)	9(75.0%)	17(81.0%)	7(58.3%)	
Female, n (%)	3(25.0%)	4(19.0%)	5(41.7%)	
DMFS, median (IQR)	2.5(2.0-4.0)	2.0(1.0-3.0)	1.0(1.0-4.5)	0.377^2^
dmfs, median (IQR)	2.5(1.75-3.25)	2.5(1.25-3.75)	2.0(1.25-2.0)	0.495^2^
PLI, median (IQR)	1.24(0.91-1.39)	1.10(0.56-1.38)	0.75(0.31-1.16)	0.145^2^

^1^Test by Chi-Squared Test, ^2^Test by Kruskal-Wallis H test. IQR, Interquartile range; AAP, Alveolar cleft adolescents’ plaque; PAP, post-operative alveolar bone grafting adolescents’ plaque; CAP, healthy control adolescents’ plaque.

**Table 2:** Descriptive statistics and statistical comparisons between AYP, PYP, and CYP.

	AYP	PYP	CYP	*p*-value
Age, median (IQR)	22(21-25)	22(20-23)	23(19.5-26.5)	0.722^3^
Gender				0.996^1^
male, n (%)	9(45.0%)	6(46.2%)	4(44.4%)	
Female, n (%)	11(55.0%)	7(53.8%)	5(55.6%)	
DMFS, median (IQR)	5.0(3.3-6.0)	4.0(3.5-5.5)	4.0(2.5-4.0)	0.088^3^
PLI, mean ± SD	0.82 ± 0.40	0.70 ± 0.33	0.61 ± 0.31	0.338^2^

^1^Test by Chi-Squared Test, ^2^Test by one-way ANOVA, ^3^Test by Kruskal-Wallis H test. IQR, Interquartile range; AYP, Alveolar cleft young adults’ plaque; PYP, post-operative alveolar bone grafting young adults’ plaque; CYP, healthy control young adults’ plaque.

**Figure 2:**
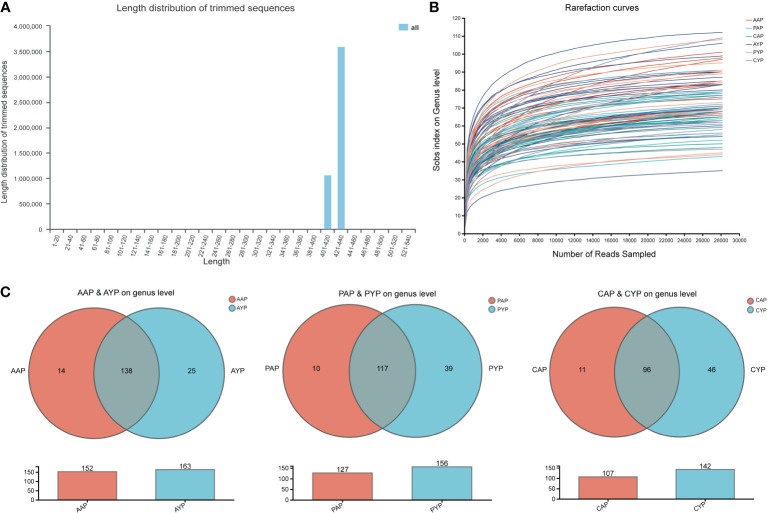
General characteristics of sample sequencing: **(A)** Distribution of the sequence length; **(B)** The Rarefaction curves at the genus level based on the Sobs index; **(C)** The similarity and overlap with the adolescent and the young adult microbiota. Venn diagram showing all the overlapping genera found in the AAP vs. AYP groups, the PAP vs. PYP group, and the CAP vs. CYP groups. The plaque microbiomes of adolescents and young adults are colored in red and blue, respectively.

We acquired a total of 4490011 optimized sequences and 1897772139 bases for further investigation after quality trimming and chimera testing. The average length of a sequence is 422 bp (**Figure 2A**). The logic of the sequencing depth and amount was further confirmed by the flattening rarefaction curves based on genus level (**Figure 2B**) and species level (**Supplementary Figure S1B**). These findings were further supported by core taxon curves (**Supplementary Figure S1A**). As a result, the measurements obtained in the samples were regarded as progressive and reasonable with an appropriate degree of taxonomy and composition. These 87 plaque samples yielded 14 phyla, 29 classes, 72 orders, 118 families, 214 genera, 469 species, and 1507 operational taxonomic units (OTUs) of plaque microbes.

*Firmicutes*, *Actinobacteriota*, *Fusobacteriota*, *Proteobacteria*, and *Bacteroidota* are the top five phyla. *Streptococcus*, *Leptotrichia*, *Actinomyces*, *Veillonella*, and *Neisseria* are the top five genera. All of the groupings are depicted at the phylum and genus levels (**Supplementary Figures S1C**, **S2**). The genus-level Venn plots show similarity and overlap between the two age groups of the three groups, with genera more abundant in young adults (**Figure 2C**).

### 3.2 Alpha diversity of dental plaque microbiota in AC patients

Chao, Ace, Shannon, and Simpson indices at the genus level were used to analyze differences in alpha diversity between three groups in two age classes. The richness of the plaque communities was assessed by Chao and Ace indices. The diversity of the plaque communities was assessed by Shannon and Simpson indices.

**Figure 3:**
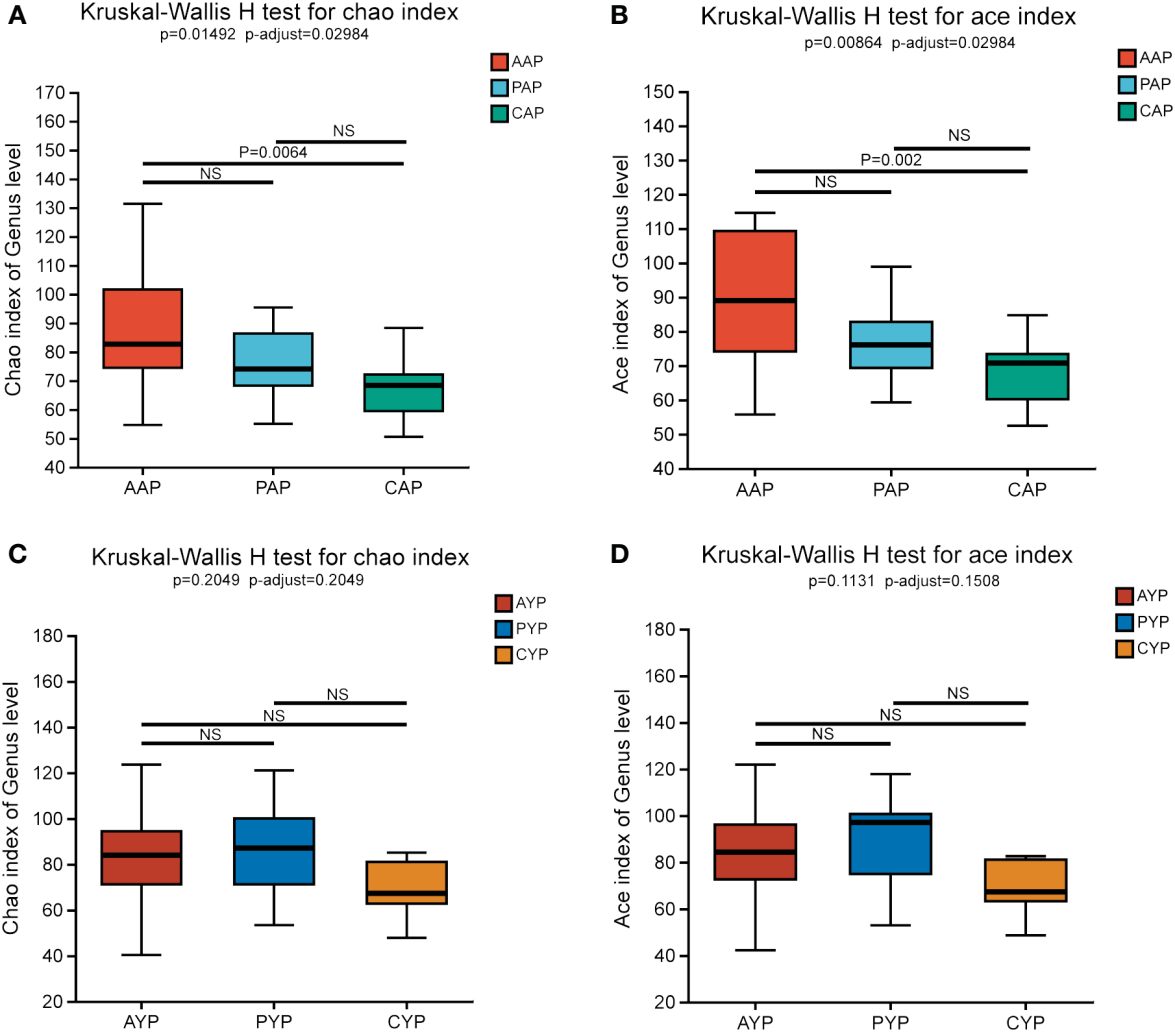
Comparing Chao Index **(A)** and Ace Index **(B)** in AAP, PAP, and CAP. Comparing Chao **(C)** and Ace Index **(D)** in AYP, PYP and CYP. Data are expressed as median (25th, 75th percentile) (Kruskal-Wallis H test, multiple comparisons: false discovery rate (FDR), *post hoc* test: Scheffe). NS, no significance.

At the age of adolescence, the Chao and Ace indices showed the same results, with AAP showing statistical differences from the healthy control CAP, and there was no statistical difference between AAP vs. PAP and PAP vs. CAP (**Figures 3A, B**). For Shannon and Simpson indices, there were no statistical differences between the three groups (**Supplementary Figures S3A, B**). In young adults, there were no significant differences between the three groups for Chao, Ace index (**Figures 3C, D**) or Shannon, Simpson index (**Supplementary Figures S3C, D**).

Overall, the difference in community richness between the adolescent alveolar cleft group and the healthy control group was more pronounced, and alveolar bone grafting did not alter plaque community richness and community diversity in alveolar cleft patients in either age group.

### 3.3 Beta diversity of dental plaque microbiota in AC patients

**Figure 4:**
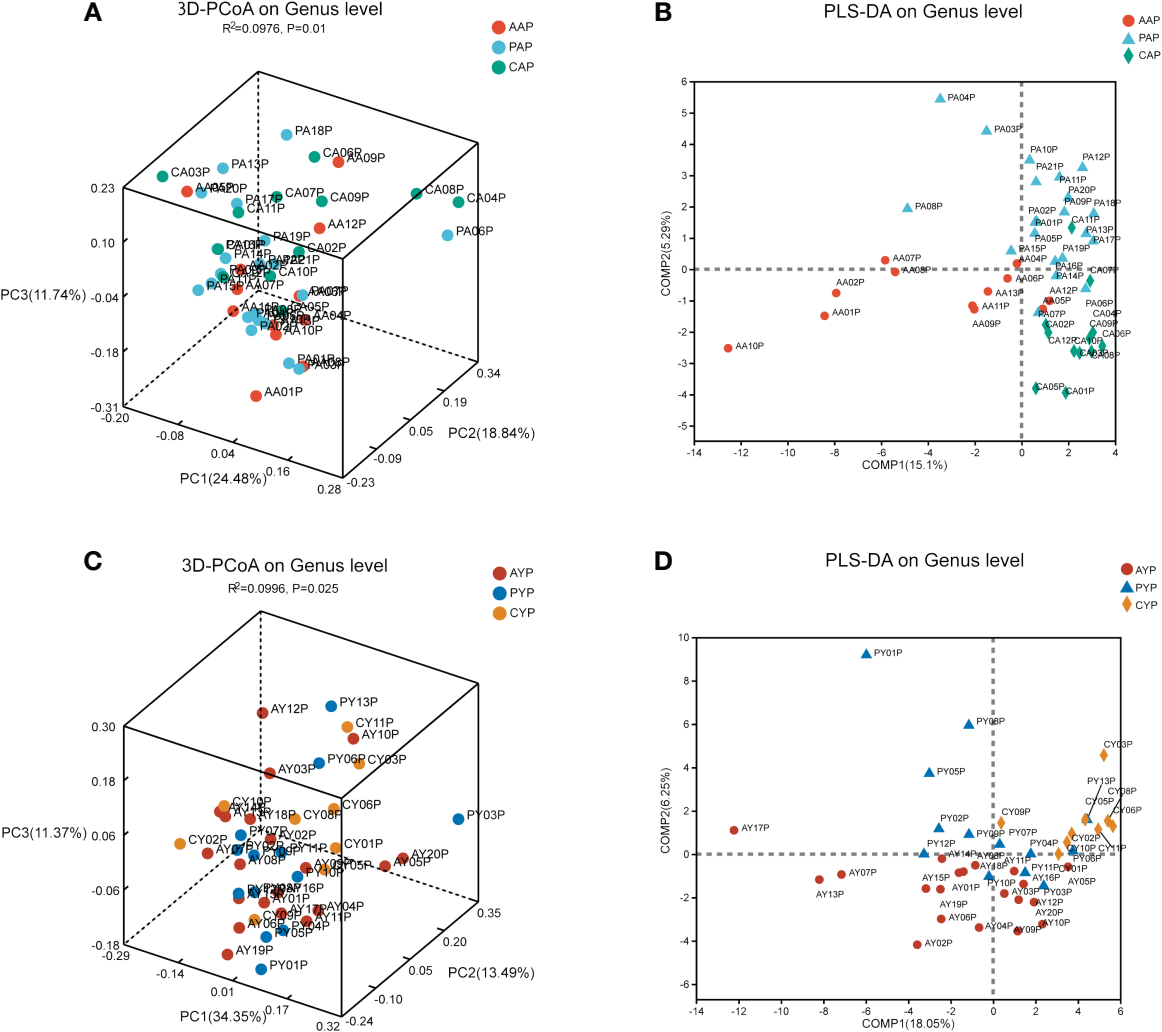
The beta diversity analysis of the microbiota of adolescents **(A)** and young adults **(C)** among the three groups. (The PCoA is based on the weighted-normalized-unifrac metric of the beta diversity analysis. Adonis tests, R² indicates the degree to which this grouping method explains the differences between samples. PCoA, Principal Coordinate Analyses). PLS-DA analysis showed the compositional differences between the bacterial communities of AAP, PAP, and CAP **(B)** and those of AYP, PYP, and CYP **(D)**.

PCoA analysis was used to examine differences in beta diversity at the genus level based on the weighted-normalized-unifrac distance algorithm to determine if there were diversity differences in microbial community composition. Between AAP, PAP, and CAP, there were significant differences in the beta diversity of the plaque microbiota (**Figure 4A**, PCoA, weighted-normalized-unifrac metric, Adonis, R-square = 0.0976, *p*-value = 0.01). Additionally, there were substantial differences in beta diversity across AYP, PYP, and CYP (**Figure 4C**, PCoA, weighted-normalized-unifrac metric, Adonis, R-squared = 0.0996, *p*-value = 0.025). The samples of the three groups in the two age classes were likewise significantly separated by PLS-DA (**Figures 4B, D**).

### 3.4 Analysis of the microbial community composition of dental plaque in AC patients

**Figure 5:**
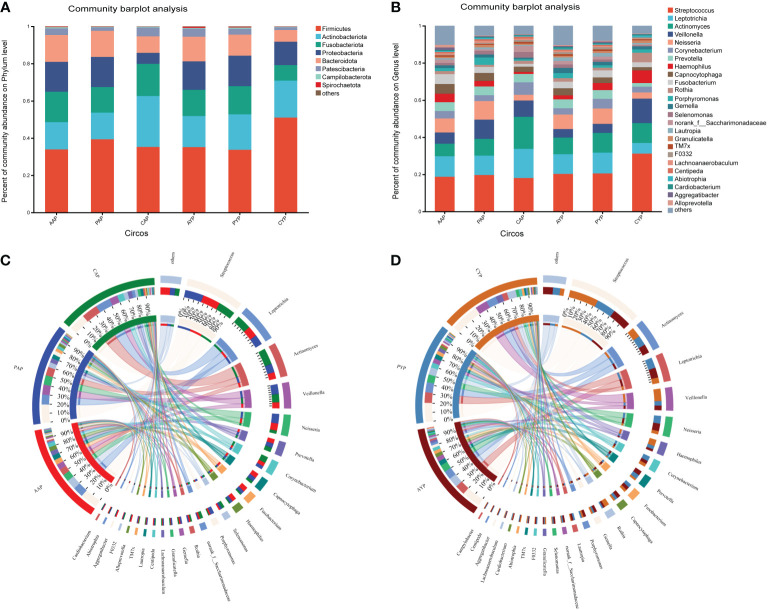
**(A)** The comparison of the bacterial composition in the six groups at the phylum level. **(B)** The comparison of the bacterial composition in the six groups at the level of genus. The circos shows the relative abundance composition of dental plaque microorganisms in three groups of adolescents **(C)** and young adults **(D)** at the genus level.

The microbiota of three groups of plaque at two ages was characterized using the relative abundance of bacteria at the phylum level (**Figure 5A**) and genus level (**Figure 5B**). The Circos plots show the composition of the plaque microbiome in a different way (**Figures 5C, D**). Compared to healthy controls, the dental plaque microbiota’s overall composition altered in AC patients and post-ABG patients at various levels of categorization in both age groups (detailed information on the composition of the microbiota at the genus level is shown in **Supplementary Figure S4**).

### 3.5 Differential analysis of plaque microbial community in AC patients

**Figure 6:**
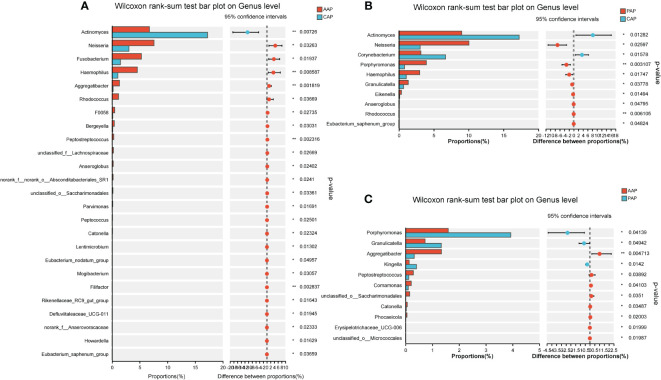
Differential analysis of bacterial genus levels in dental plaque between two groups of adolescents. **(A)** Differential analysis at genus level between AAP and CAP groups. **(B)** Differential analysis at genus level between PAP and CAP groups. **(C)** Differential analysis at genus level between AAP and PAP groups. *p < 0.05, **p < 0.01.

The microbial composition at the genus level was analyzed for differences between the two age groups. For the adolescent groups, a total of 25 bacterial genera were identified with statistical differences in abundance between the AAP and CAP groups (**Figure 6A**). Meanwhile, there were 10 bacterial genera with statistical differences in abundance between the PAP and CAP groups (**Figure 6B**), and 6 of these 10 bacterial genera were the same as the differential genera in AAP vs. CAP. They are *Actinomyces, Neisseria, Haemophilus, Anaeroglobus, Rhodococcus*, and *Eubacterium*.

**Figure 7:**
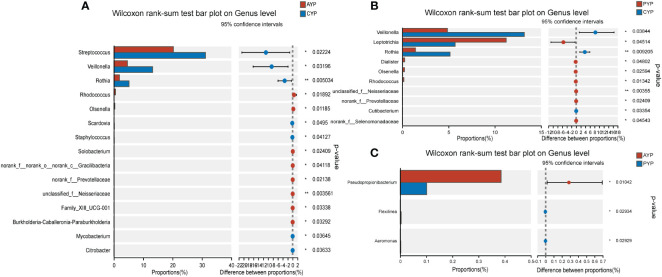
Differential analysis of bacterial genus levels in dental plaque between two groups of young adults. **(A)** Differential analysis at genus level between AYP and CYP groups. **(B)** Differential analysis at genus level between PYP and CYP groups. **(C)** Differential analysis at genus level between AYP and PYP groups. *p < 0.05, **p < 0.01.

Interestingly, for the young adults, a total of 15 bacterial genera were identified with statistical differences in abundance between the AYP and CYP groups (**Figure 7A**). Meanwhile, there were also 10 bacterial genera with statistical differences in abundance between the PYP and CYP groups (**Figure 7B**), and also 6 of these 10 bacterial genera were the same as the differential genera in AYP vs. CYP. They are *Veillonella, Rothia, Olsenella, Rhodococcus, unclassified*_*f*_*Neisseriaceae*, and *norank*_*f*_*Prevotellaceae*. These show the effect of the once-alveolar cleft stage on the PAP and PYP groups. And there was a decrease in differential bacterial genera in the post-ABG groups compared to the healthy controls than the AC groups.

Comparisons between patients with alveolar clefts and patients after alveolar bone grafting showed 11 bacterial genera were identified with statistical differences in abundance between AAP and PAP (**Figure 6C**), 3 bacterial genera were identified with statistical differences in abundance between AYP and PYP (**Figure 7C**), and a greater effect of alveolar bone grafting on plaque in patients with alveolar clefts at the adolescent stage.

**Figure 8:**
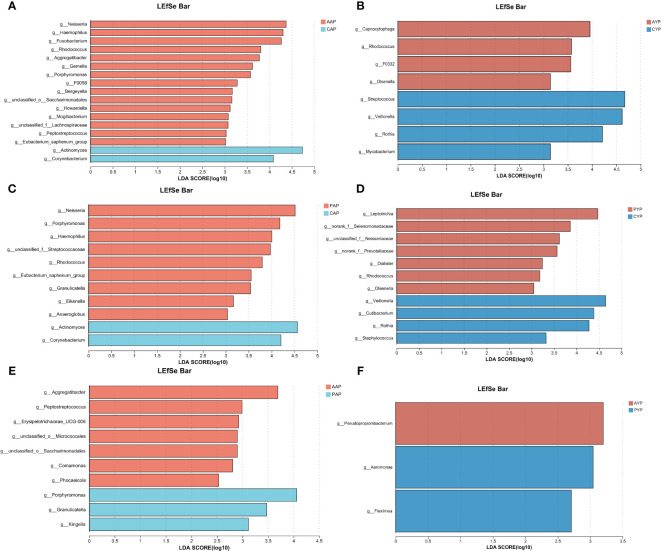
A logarithmic LDA score cutoff was used to identify significant genera differences between the plaque microbiomes of the two groups. **(A)** Significantly enriched genera between the AAP and CAP groups as determined by LEfSe analysis (LDA score > 3). **(B)** Significantly enriched genera between the AYP and CYP groups as determined by LEfSe analysis (LDA score > 3). **(C)** Significantly enriched genera between the PAP and CAP groups as determined by LEfSe analysis (LDA score > 3). **(D)** Significantly enriched genera between the PYP and CYP groups as determined by LEfSe analysis (LDA score > 3). **(E)** Significantly enriched genera between the AAP and PAP groups as determined by LEfSe analysis (LDA score > 2.5). **(F)** Significantly enriched genera between the AYP and PYP groups as determined by LEfSe analysis (LDA score > 2.5). LEfSe, an effect size of the linear discriminant analysis (LDA).

Next, we used linear discriminant analysis (LDA) effect sizes (LEfSe) to identify genus-level differences between two groups (LDA scores > 3, *p* < 0.05), and log LDA score cutoffs of 3.5 to identify important genus-level differences. When compared to the corresponding health controls, *Neisseria, Haemophilus, Fusobacterium, Rhodococcus, Aggregatibacter, Gemella, and Porphyromonas* are the dominant genera in the AAP group (**Figure 8A**). *Neisseria, Porphyromonas, Haemophilus, Rhodococcus*, *Eubacterium*, *and Granulicatella* are the dominant genera in the PAP group (**Figure 8C**). *Capnocytophaga, Rhodococcus*, and *F0332* are the dominant genera in the AYP group (**Figure 8B**). And *Leptotrichia, norank_f_Selenomonadaceae, unclassified*_*f*_*Neisseriaceae, norank*_*f*_*Prevotellaceae* are the dominant genera in the PYP group (**Figure 8D**).

The impact of alveolar bone grafting on AC patients was examined in more detail, and we found differences between AC patients and post-ABG patients (LDA score > 2.5, *p* < 0.05), and log LDA score cutoffs of 3.0 to identify important genus-level differences. *Porphyromonas, Granulicatella*, and *Kingella* are more prevalent in the PAP group (**Figure 8E**), and *Aeromonas* is the dominant genus in PYP group (**Figure 8F**).

### 3.6 Phenotypic differences in plaque microbiota of AC patients

**Figure 9:**
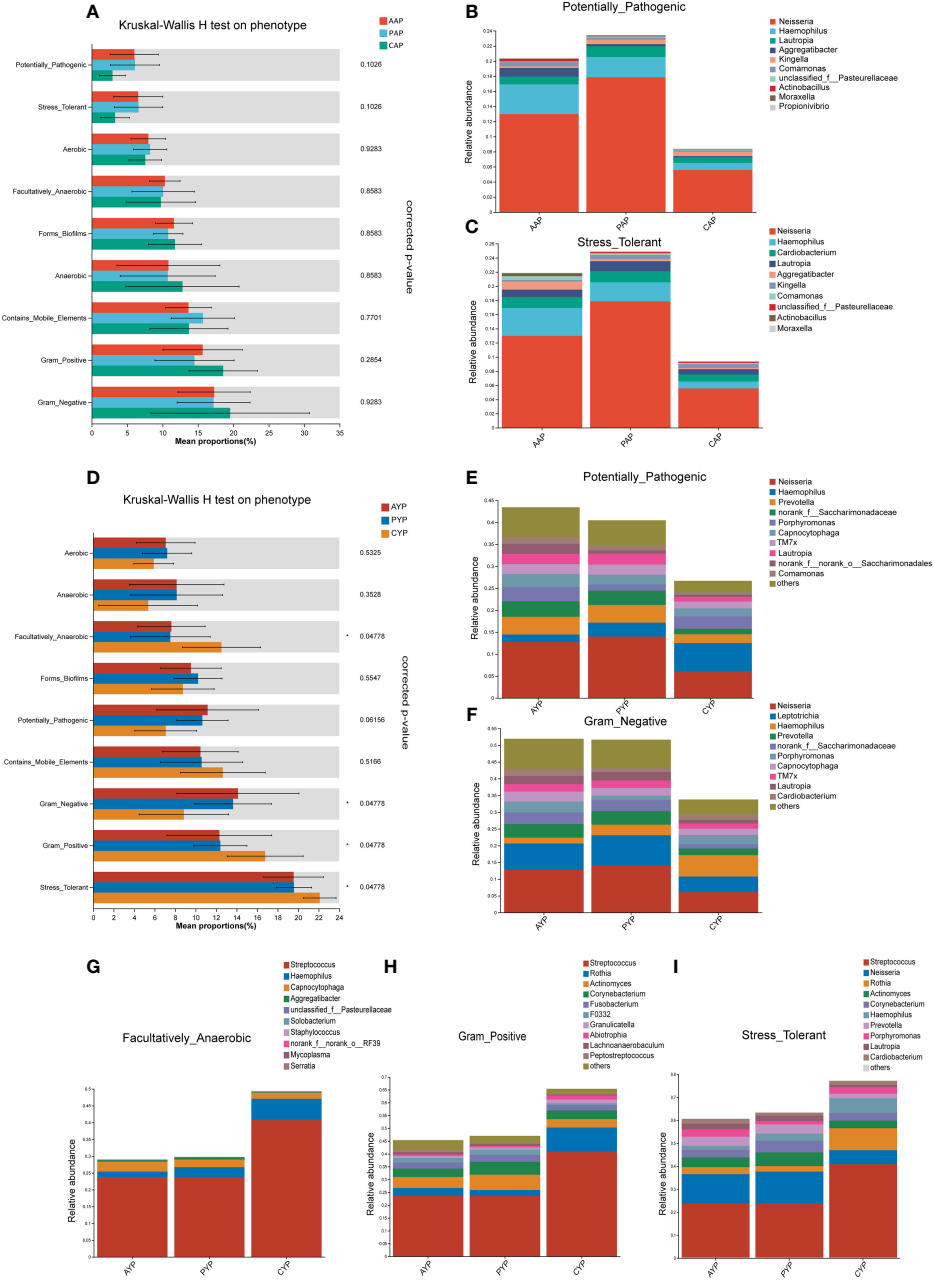
Prediction of microbiota phenotypic function in adolescents **(A)** and young adults **(D)** with alveolar clefts at the genus level. The relative abundance of microbial potentially pathogenic phenotypes **(B)** and stress tolerance phenotypes **(C)** in three groups of adolescents. The relative abundance of microbial potentially pathogenic phenotypes **(E)**, Gram-negative phenotypes **(F)**, facultatively anaerobic phenotypes **(G)**, Gram-positive phenotypes **(H)**, and stress tolerance phenotypes **(I)** in three groups of young adults. Mean Proportion%: The percentage of the mean relative abundance. *FDR-corrected *p* < 0.05.

Phenotypic prediction was performed using BugBase. Among the adolescents (**Figure 9A**), there was no statistically significant difference between the potentially pathogenic phenotype (*p* = 0.0228, corrected-*p* = 0.1026, FDR) and the stress-tolerant phenotype (*p* = 0.0138, corrected-*p* = 0.1026, FDR) in the Kruskal-Wallis H test for three groups. However, according to the relative abundance of microbial phenotypes, there was a higher expression of potentially pathogenic phenotypes (**Figure 9B**) and stress-tolerant phenotypes (**Figure 9C**) in the AAP and PAP groups compared to the CAP group, which was associated with an increase in the relative abundance of *Neisseria*, *Haemophilus*, *Lautropia*, *Aggregatibacter*, *Comamonas*, *Actinobacillus*, *Moraxella* and *Cardiobacterium*.

Between the three groups of young adults (**Figure 9D**), there was a significant difference in the four phenotypes facultatively anaerobic, Gram-negative, Gram-positive, and stress-tolerant (corrected-*p* < 0.05, FDR), and no statistically significant difference in the potentially pathogenic phenotype (*p* = 0.0342, corrected-*p* = 0.061, FDR). While in terms of the relative abundance of microbial phenotypes, there was a higher expression of potentially pathogenic phenotype (**Figure 9E**) and Gram-negative phenotype (**Figure 9F**) in the AYP and PYP groups compared to the CYP group, which was associated with an increase in the relative abundance of *Neisseria*, *Prevotella*, *norank*_*f*_*Saccharimonadaceae*, *Capnocytophaga*, *TM7x*, *Lautropia*, *Comamonas*, *Leptotrichia*. In addition, the AYP and PYP groups exhibited reduced expression of the facultatively anaerobic phenotype (**Figure 9G**), Gram-positive phenotype (**Figure 9H**), and stress-tolerant phenotype (**Figure 9I**) compared to CYP, which was mainly associated with a reduction in *Streptococcus* abundance at the genus level. Meanwhile, there were no statistically significant differences in phenotype prediction between the AAP vs. PAP and the AYP vs. PYP.

## 4 Discussion

The investigation of oral microbiota necessitates the integration of temporal, geographical, and functional factors in conjunction with composition and structure (Proctor et al., 2020). Integral dysbiosis, an imbalance in the makeup of the microbiota, is intimately associated with the onset of certain diseases and significantly undermines overall well-being (Solbiati and Frias-Lopez, 2018). In the interim, modifications in the composition of the oral microbiota have been documented in numerous disorders, including heart failure (Yuzefpolskaya et al., 2023), cardiovascular disease (Altamura et al., 2023), Alzheimer’s disease (Loughman et al., 2023), oral cancer (Pispero et al., 2022), type 2 diabetes (Guo et al., 2023), rheumatoid arthritis (Afrasiabi et al., 2023), and systemic lupus erythematosus (Graves et al., 2019). Therefore, it is imperative to investigate the modifications that environmental variables and pathogens produce in oral microbiota. Dental plaque consists of a complex community of microorganisms, and this typical biofilm is the etiologic agent of oral diseases such as caries and periodontal disease (Seneviratne et al., 2011). A significant negative correlation ratio between nodes of the caries microbial network indicates severe competition among microorganisms and leads to the network’s enhanced complexity and interaction intensity (Shao et al., 2023).

According to the biogeography of microorganisms, taxon distributions and spatial variation within ecosystems are significant determinants of ecological communities, and these environmental characteristics affect the structure and function of bacterial communities (Mccallum and Tropini, 2023). Previous microbiome studies of cleft lip and palate have focused on infants and children who have not undergone cleft lip and palate repair, and limited attention has been paid to the time frame around alveolar clefts and post-alveolar bone grafting. It is crucial to illustrate the oral microbiota during this era to better comprehend the causes of the distinctive oral condition of patients with AC, as oral issues are prevalent among adolescents with AC. In addition, numerous young adult patients seek counseling for ABG surgery due to financial and educational considerations. To compare these two age groups with age-matched healthy controls, we incorporated adolescents and young adults who were patients with AC and those who had undergone ABG treatment.

The LEfSe analysis revealed that *Neisseria, Haemophilus, Fusobacterium, Rhodococcus, Aggregatibacter, Gemella*, and *Porphyromonas* exhibited a significant abundance among adolescent patients diagnosed with AC. Variations in the amount of *Capnocytophaga* and *Rhodococcus* at the genus level were particularly pronounced in young adult patients with AC who have had the condition for a longer period of time. A recent study identified *Neisseria* and *Gemella* as oral-nasal translocation bacteria, as some of the bacterial biomarkers in the nasal cavity of cleft palate patients were common oral flora, suggesting that bacterial translocation between the oral and nasal niches may occur in cleft palate patients. And the nasal bacterial network is composed of the following genera: *Streptococcus, Gemella, Alloprevotella, Neisseria, Rothia, Actinomyces, and Veillonella*, according to this study (Zhou et al., 2022). Notably, the most abundant sequences at the genus level in a study sequencing microorganisms isolated from tonsil crypts and adenoid surfaces were *Fusobacterium, Haemophilus, and Neisseria* (Johnston et al., 2019); These genera are comparable to the differential genera of AAP vs. CAP and PAP vs. CAP found in our study.

When analyzing differences in genus composition, for adolescents, *Actinomyces, Neisseria, Haemophilus, Anaeroglobus, Rhodococcus*, and *Eubacterium* were the most common differential genera for AAP vs. CAP and PAP vs. CAP. For young adults, *Veillonella, Rothia, Olsenella, Rhodococcus, unclassified*_*f*_*Neisseriaceae*, and *norank*_*f* ***_*** *Prevotellaceae* were the most common differential genera for AYP vs. CYP and PYP vs. CYP. These further demonstrated the effect of the period of previous alveolar cleft on plaque in post-ABG patients. Meanwhile, the post-ABG vs. healthy control group showed fewer differences in bacterial genera at both ages than the AC vs. healthy control group.

Following this, an analysis was conducted on the alterations in plaque microbiota-associated functional phenotype in healthy controls, patients with AC, and post-operative ABG patients. For young adults, evaluation of the BugBase data revealed a significant difference in the facultatively anaerobic, Gram-negative, Gram-positive, and stress-tolerant phenotypes between AYP and CYP. However, there was no statistically significant difference in phenotypic function between AAP and CAP. Functional changes in the plaque were more pronounced in the young adult group with a longer duration of alveolar clefts. Meanwhile, according to the relative abundance of microbial phenotypes, an increase in *Neisseria* was present at both ages and positively influenced the respective potential pathogen phenotypes. *Neisseria*, classified in the phylum *Proteobacteria*, is a prevalent microbe seen in early childhood caries (ECC) (Xu et al., 2023) and is crucial in the progression of dental caries (Dinis et al., 2022).

In general, alien species are unable to invade or colonize the microbial flora in the oral cavity. In patients with alveolar clefts, this ability is hampered, however, as a consequence of the anatomical and morphological anomalies that are linked to these conditions, resulting in oral disease (Wu et al., 2022). In patients with cleft lip and palate from infancy, the abnormal contact between the nasopharynx and the oral cavity makes these two malformed cavities more susceptible to abnormal development of the flora. Even if the cleft lip and palate were repaired in infancy, the alveolar cleft persists into adolescence, along with skeletal deformity, and the child’s inability to control oral hygiene increases the incidence of dental caries, periodontal disease, and upper respiratory tract infections (Gershater et al., 2023). In addition, co-infection with various opportunistic/pathogenic bacterial strains is a potentially important risk factor for local and systemic disease in alveolar cleft patients (Arboleda et al., 2023). Dental caries is a predominant plaque-mediated illness affecting the oral cavity. In recent years, our understanding of the mechanisms underlying dental caries has shifted from the specific plaque theory to the oral microbiome (Takahashi, 2023). The ecological plaque hypothesis and dysbiotic communities ultimately succinctly encapsulate the underlying principles of the oral microbiome (Belibasakis et al., 2023). Thus, the emergence of oral disorders such as caries is not attributable to a particular bacteria but rather to a transformation in the organization of the microbial community encompassing all oral microorganisms. The relative abundance of bacterial genera in patients with alveolar clefts differed significantly both numerically and phenotypically in our study.

It is noteworthy that Venn diagram analysis of plaque at the genus level revealed a progressive increase in the number of distributions for the three groups at both ages in the AAP-AYP, PAP-PYP, and CAP-CYP groups. The increase in bacterial operational taxonomic units (OTUs) in infant saliva from an average of 31 OTUs at 1.9 months to 84 OTUs at 39 months may provide a comparable explanation (Dashper et al., 2019). The oral microbiota undergoes expansion with age (Xu et al., 2018). As adolescents and young adults advance in age, there is variability in the number of oral bacteria present in plaque. Therefore, our research was segmented into two age cohorts, including, not to be overlooked, the young adult patients with AC who had not received an ABG.

Regarding to clinical parameters of periodontal disease in individuals with CLP, a meta-analysis showed a significant difference in mean plaque index scores, gingival index scores, and periodontal pocket depth between individuals with and without CLP (Marzouk et al., 2022). And on the effect of ABG on periodontal conditions, a study of periodontal health differences between the cleft side and non-cleft side nearly a decade after secondary alveolar bone grafting, there was no significant difference in probing pocket depth, few of pathological gingival pockets, but the teeth on the cleft side had high levels of gingival inflammation (Lemberger et al., 2024). Therefore, our study has some research limitations that need to be acknowledged. First, the study did not measure periodontal pockets and other parameters of periodontal disease when considering changes in plaque bacteria. Second, since this was a single-center research with a small sample size, further multicenter studies are needed to expand the sample size. Third, although 16S rRNA sequencing can recognize the classification and composition of the dental plaque microbiome, it has limitations in identifying species and strains and does not provide direct data on functionally precise changes in the microbiome. These limitations need to be improved in our future studies.

## 5 Conclusion

Our study systematically characterized the supragingival dental plaque microbiota of alveolar cleft patients, post-alveolar bone grafting patients, and matched healthy controls at two ages to improve the understanding of the oral ecology and microbiology associated with alveolar clefts. Second, we found differences in the composition of their dental plaque microorganisms between the alveolar cleft group and the healthy control group at both ages, and the bacteria associated with alveolar clefts and potential biomarkers identified by LEfSe analysis. Alveolar cleft patients may have an impact on plaque flora due to their anatomical structure. And there were some differences in functional phenotypes between the young adult alveolar cleft group with a longer period of alveolar cleft and the healthy control group. Meanwhile, there was no statistically significant difference in the functional phenotype of dental plaque between preoperative and postoperative patients with alveolar bone grafts at either age, indicating that alveolar bone grafting did not alter the phenotypic function of dental plaque in individuals with alveolar clefts. However, when compared with healthy controls, the post-ABG groups had smaller differences than the AC groups at two ages, and the amount of genus difference reduction was more pronounced in adolescents.

## Data availability statement

The datasets presented in this study can be found in online repositories. The names of the repository/repositories and accession number(s) can be found below: NCBI, accession number PRJNA1062077.

## Ethics statement

The studies were approved by the Ethics Committee of Shanghai Ninth People's Hospital, Shanghai Jiao Tong University School of Medicine. Written informed consent to participate in this study was obtained from the legal guardians/participants themselves.

## Author contributions

YZ: Conceptualization, Data curation, Investigation, Methodology, Software, Visualization, Writing – original draft, Writing – review & editing. QZ: Conceptualization, Formal analysis, Investigation, Software, Writing – original draft, Writing – review & editing. JS: Conceptualization, Data curation, Investigation, Methodology, Writing – original draft, Writing – review & editing. ZJ: Conceptualization, Investigation, Validation, Writing – original draft, Writing – review & editing. ZZ: Formal analysis, Project administration, Supervision, Validation, Writing – original draft, Writing – review & editing. ZC: Conceptualization, Formal analysis, Funding acquisition, Project administration, Resources, Supervision, Writing – original draft, Writing – review & editing.
