# Supplementary material for: Characterization and functional prediction of the dental plaque microbiome in patients with alveolar clefts
Source: Front Cell Infect Microbiol. 2024 May 10;14:1361206. doi: 10.3389/fcimb.2024.1361206 (PMC11119321; doi:10.3389/fcimb.2024.1361206)
Supplement: Supplementary file 1 [file DataSheet_1.zip › supplementary material(0411)/supplementary figure and table(0411).DOCX]

Supplementary Material


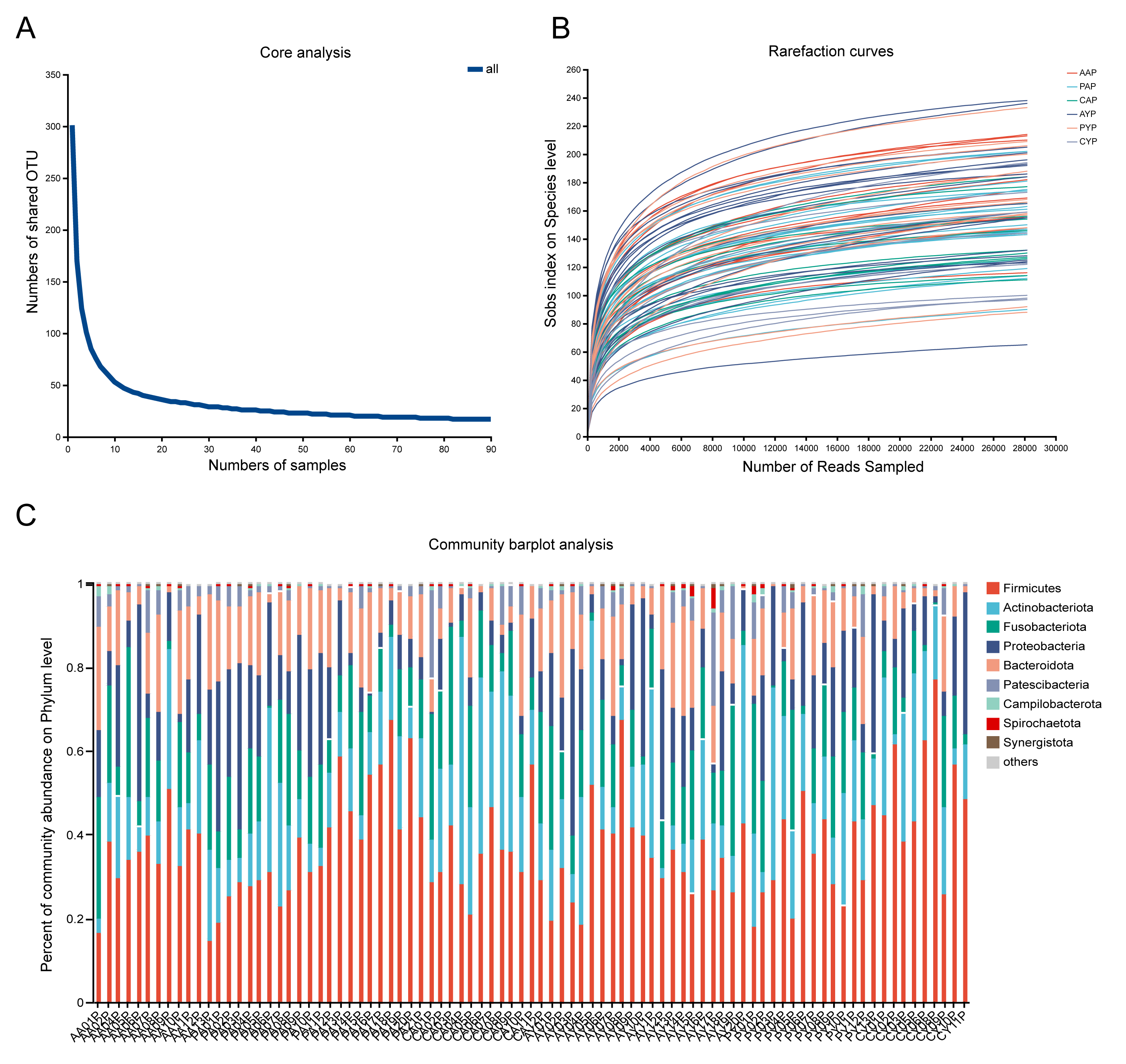


**Supplementary Figure S1.** Basic information on sequencing.

**Supplementary Figure S2.** Percent of community abundance on genus level for each sample.


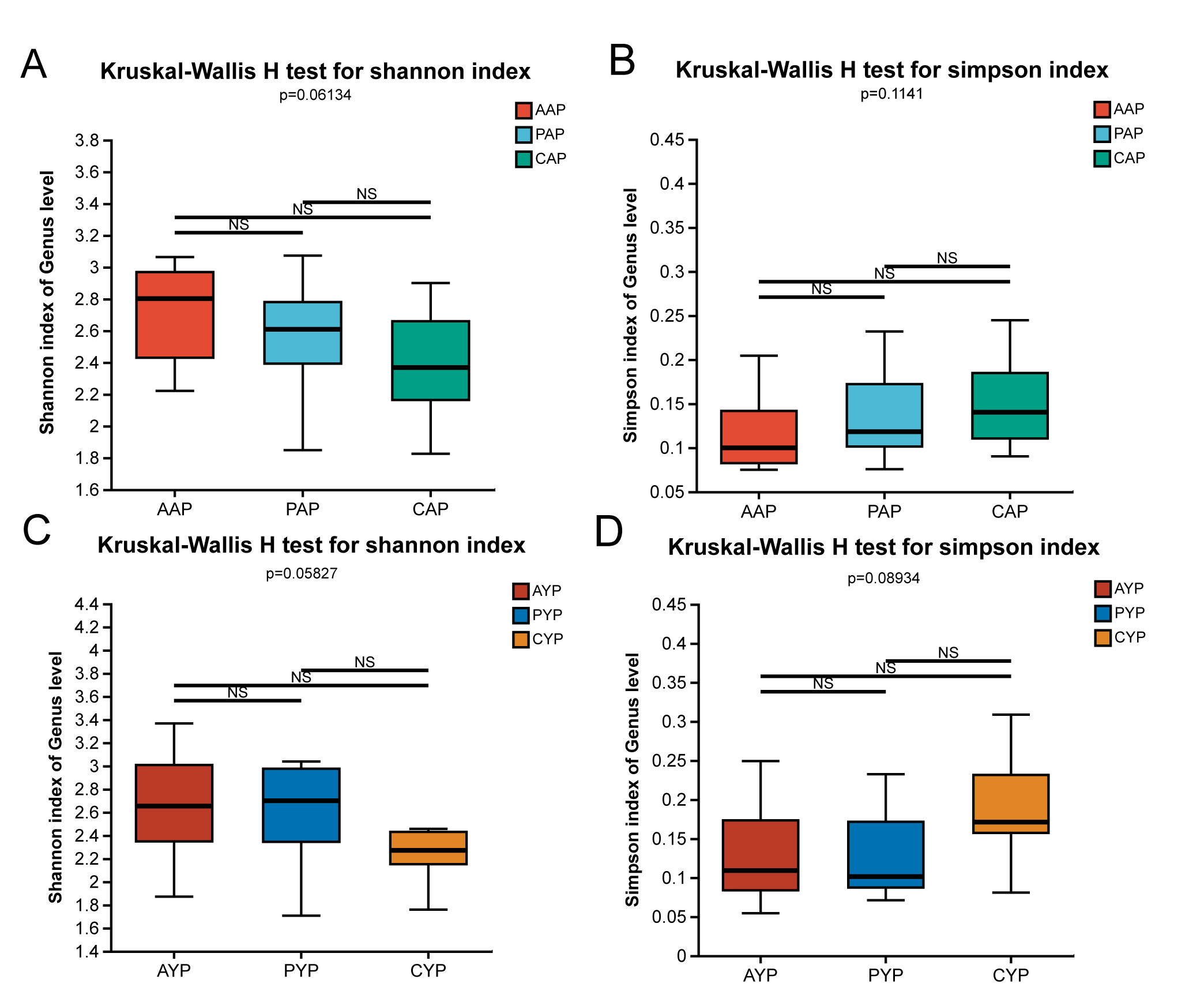


**Supplementary Figure S3.** Comparison of Shannon Index (A) and Simpson Index (B) in AAP, PAP and CAP. Comparison of Shannon Index (C) and Simpson Index (D) in AYP, PYP and CYP.


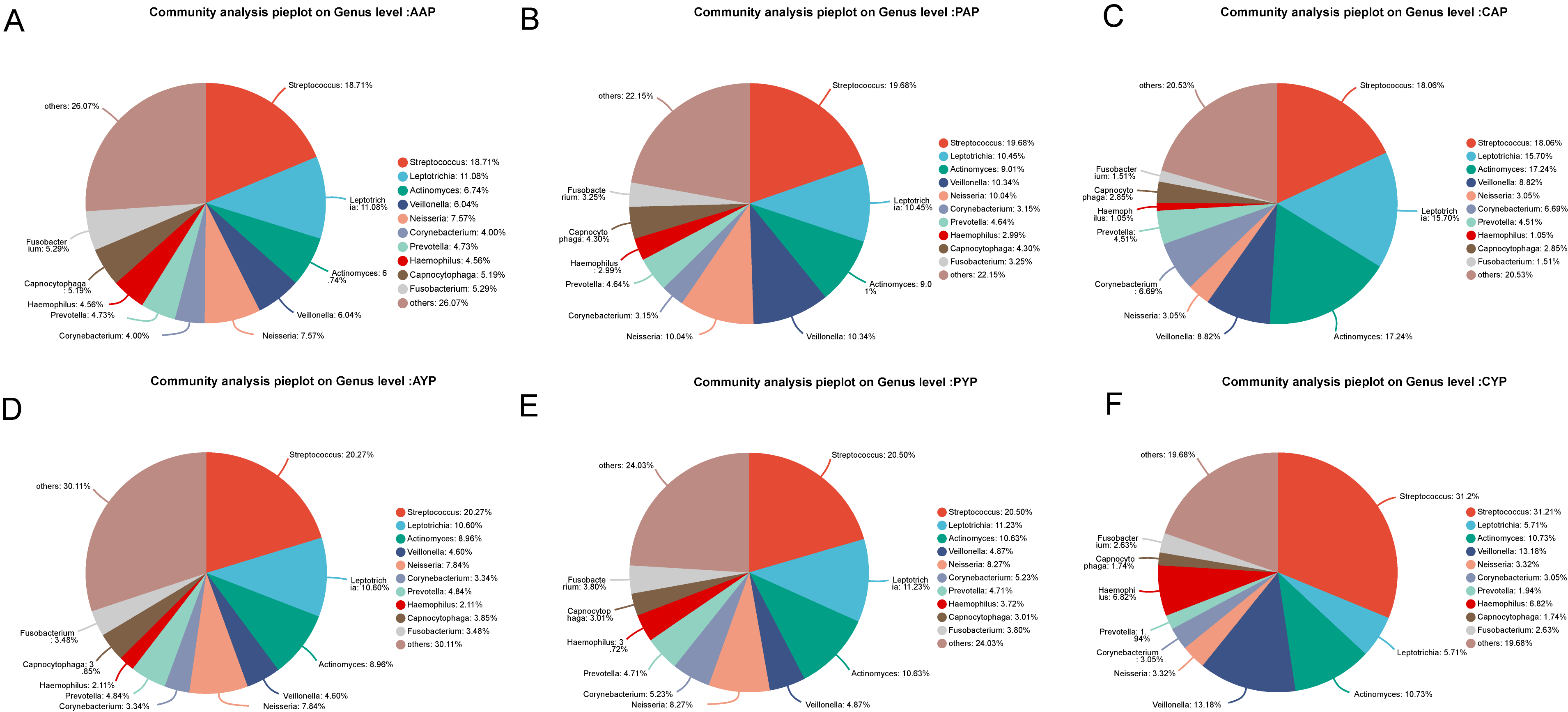


**Supplementary Figure S4.**The community pieplot on the genus level of each group.

**Supplementary Table S1.Basic information of included adolescents.**

| Number | Namer | Group | Age | Gender | DMFS | dmfs | PLI |
| --- | --- | --- | --- | --- | --- | --- | --- |
| 1  2  3  4  5  6  7  8  9  10  11  12  13  14  15  16  17  18  19  20  21  22  23  24  25  26  27  28  29  30  31  32  33  34  35  36  37  38  39  40  41  42  43  44  45 | AA01P  AA02P  AA04P  AA05P  AA06P  AA07P  AA08P  AA09P  AA10P  AA11P  AA12P  AA13P  PA01P  PA02P  PA03P  PA04P  PA05P  PA06P  PA07P  PA08P  PA09P  PA10P  PA11P  PA12P  PA13P  PA14P  PA15P  PA16P  PA17P  PA18P  PA19P  PA20P  PA21P  CA01P  CA02P  CA03P  CA04P  CA05P  CA06P  CA07P  CA08P  CA09P  CA10P  CA11P  CA12P  *p*-value | 1  1  1  1  1  1  1  1  1  1  1  1  2  2  2  2  2  2  2  2  2  2  2  2  2  2  2  2  2  2  2  2  2  3  3  3  3  3  3  3  3  3  3  3  3 | 12  18  15  6  17  12  16  17  16  9  12  11  14  12  17  16  8  9  9  17  8  16  8  12  13  10  11  11  15  8  16  10  17  8  14  12  9  14  11  12  15  14  12  12  10  0.409^2^ | M  F  M  M  M  F  M  M  M  M  F  M  M  M  M  M  M  F  M  M  F  M  M  F  M  M  M  M  M  M  M  F  M  M  M  M  F  F  F  M  M  F  M  M  F  0.364^1^ | 2  4  5  2  4  1  2  5  3  3  2  1  7  2  6  3  1  1  2  3  2  2  2  3  2  3  2  2  4  0  1  1  1  1  3  1  1  5  2  1  6  5  1  1  1  0.377^2^ | 3  2  2  4  3  1  2  4  2  3  2  3  5  3  5  1  1  1  2  2  2  2  2  1  2  4  0.495^2^ | 1.39  1.00  0.88  1.10  0.44  1.20  1.38  1.39  0.55  1.29  1.39  1.27  2.00  1.22  1.38  0.55  0.83  0.78  0.50  0.16  1.22  1.10  1.33  1.44  1.38  0.44  1.67  1.39  1.11  1.06  0.56  0.61  0.55  1.11  0.94  0.28  1.39  0.38  1.11  1.17  0.55  0.27  1.28  0.55  0.27  0.145^2^ |

**^1^Test by Chi-Squared Test,^2^test by Kruskal-Wallis test.**

**Supplementary Table S2. Basic information of included young adults.**

| Number | Namer | Group | Age | Gender | DMFS | PLI |
| --- | --- | --- | --- | --- | --- | --- |
| 1  2  3  4  5  6  7  8  9  10  11  12  13  14  15  16  17  18  19  20  21  22  23  24  25  26  27  28  29  30  31  32  33  34  35  36  37  38  39  40  41  42 | AY01P  AY02P  AY03P  AY04P  AY05P  AY06P  AY07P  AY08P  AY09P  AY10P  AY11P  AY12P  AY13P  AY14P  AY15P  AY16P  AY17P  AY18P  AY19P  AY20P  PY01P  PY02P  PY03P  PY04P  PY05P  PY06P  PY07P  PY08P  PY09P  PY10P  PY11P  PY12P  PY13P  CY01P  CY02P  CY03P  CY05P  CY06P  CY08P  CY09P  CY10P  CY11P  *p*-value | 4  4  4  4  4  4  4  4  4  4  4  4  4  4  4  4  4  4  4  4  5  5  5  5  5  5  5  5  5  5  5  5  5  6  6  6  6  6  6  6  6  6 | 23  21  20  21  20  28  27  25  22  24  25  20  22  28  27  22  21  22  24  20  22  19  19  28  24  21  27  22  22  22  22  22  19  26  21  19  19  27  25  20  23  28  0.722^3^ | M  M  F  F  F  M  F  F  M  M  F  M  M  F  M  M  F  F  F  F  M  M  F  F  M  M  M  F  M  F  F  F  F  F  F  F  F  F  M  M  M  M  0.996^1^ | 5  6  5  3  6  3  6  5  4  5  5  4  3  5  6  3  5  3  6  6  4  4  5  3  8  5  4  6  3  4  6  3  5  4  3  2  4  4  4  6  3  2  0.088^3^ | 1.33  1.38  0.61  0.78  0.94  0.83  0.55  1.67  0.22  1.11  0.94  0.78  0.44  0.55  0.72  0.28  1.05  1.38  0.44  0.38  1.33  0.55  0.72  0.28  1.05  0.89  0.83  0.78  0.28  0.94  0.39  0.28  0.72  0.44  0.33  0.27  0.88  1.11  0.83  0.55  0.28  0.83  0.338^2^ |

**^1^Test by Chi-Squared Test,^2^test by one-way ANOVA,^3^Test by independent-sample**

**Kruskal-Wallis test.**
